# From Susceptible to Resistant: The Emergence of Carbapenemase-Producing Escherichia coli

**DOI:** 10.7759/cureus.109096

**Published:** 2026-05-18

**Authors:** Anish C Kadam, Harsha V Patil, Satish R Patil

**Affiliations:** 1 Department of Microbiology, Krishna Institute of Medical Sciences, Krishna Vishwa Vidyapeeth (Deemed to be University), Karad, IND

**Keywords:** antimicrobial resistance (amr), carbapenemase-producing e. coli, escherichia coli (e. coli), morbidity, mortality, multidrug resistance (mdr)

## Abstract

Carbapenemase-producing *Escherichia coli (E. coli)* has emerged as a critical contributor to antimicrobial resistance (AMR), significantly compromising the efficacy of last-resort carbapenem antibiotics. Carbapenemase-producing *E. coli* has significantly reduced the effectiveness of carbapenems, which were previously considered last-resort antibiotics for treating severe infections caused by extended-spectrum *β*-lactamase (ESBL)-producing organisms. Numerous *β*-lactam antibiotics, including carbapenems, are hydrolyzed by these enzymes, which results in fewer therapy choices, greater rates of treatment failure, and higher rates of morbidity and death.

Travel, medical tourism, globalization, and poor infection control practices contribute to the development of resistant strains. AMR spreads more quickly in nations such as India due to factors such as over-the-counter antibiotic usage, inadequate antimicrobial stewardship, and a shortage of diagnostic infrastructure. The high frequency of *E. coli* in clinical infections and its notable resistance to commonly utilized antibiotics are highlighted by surveillance data from national programs like the ICMR-AMRSN.

Both intrinsic and acquired mechanisms contribute to resistance in *E. coli*. ESBLs, *AmpC*, and carbapenemases are clinically relevant families of β-lactamases. Carbapenemases fall into three categories: Class A (KPC, for example), Class B (metallo-β-lactamases, such as New Delhi metallo-β-lactamase (NDM), Verona integron-borne metallo-β-lactamase (VIM), and Imipenemase (IMP), and Class D (OXA-type enzymes). Many of these enzymes are plasmid-mediated and capable of rapid horizontal gene transfer.

## Introduction and background

Theodor Escherich first isolated and characterized *Escherichia coli* (*E. coli*), a rod-shaped, gram-negative bacterium from the *Enterobacteriaceae* family, from infant stool in 1885. *E. coli* colonizes the gastrointestinal tract of infants within a few hours after birth. Commensal *E. coli* strains coexist with humans without causing harm due to mutual benefits. However, immunocompromised hosts or individuals with impaired gastrointestinal barriers may become infected with commensal *E. coli*. The emergence of carbapenemase-producing *E. coli* represents a major global public health crisis, shifting from susceptible to high-level resistance (up to 65% in some studies) against last-resort carbapenem antibiotics. Driven by mobile genetic elements (e.g., OXA-48, NDM-5) and high-risk clones (e.g., ST131, ST167), these pathogens exhibit multi-drug resistance [1]. *Escherichia coli* (*E. coli*) is a clinically significant bacterium due to its dual role as a common commensal and an opportunistic pathogen in both humans and animals, facilitating the transmission and evolution of antimicrobial resistance (AMR). *E. coli* plays a central role in the emergence and dissemination of AMR due to its genetic adaptability and widespread clinical presence [2].

## Review

Search strategy

**Figure 1:**
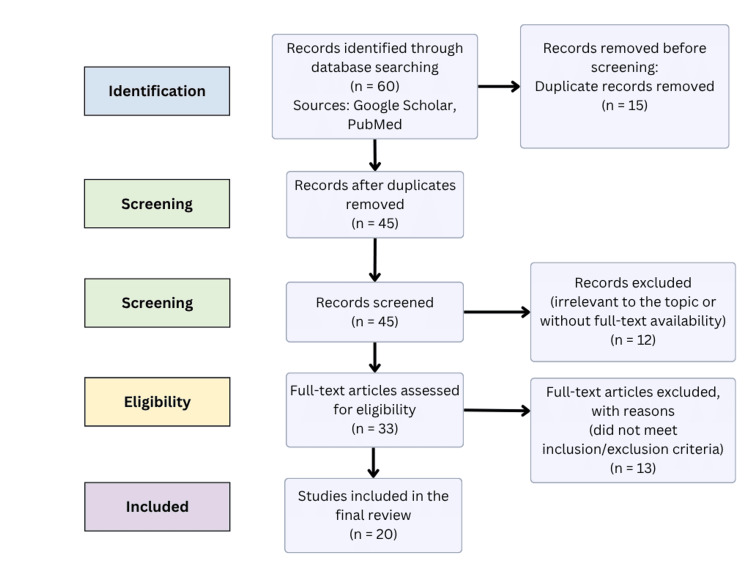
Article selection and screening process n: number of articles Image credit: This is an original image created by the authors based on a literature search strategy

A thorough literature search was conducted using databases such as Google Scholar and PubMed to develop this review on carbapenemase-resistant *Escherichia coli* (*E. coli*). MeSH terms and keywords included “*Escherichia coli*,” “carbapenemase-producing *E. coli*,” “carbapenem resistance,” “antimicrobial resistance,” “epidemiology,” and “*E. coli* infections,” combined using Boolean operators (AND, OR). Articles published in English from 2011 to 2026 were included (Figure 1). Inclusion criteria comprised original research articles, reviews, and surveillance studies focusing on carbapenemase-producing *E. coli*. Studies not available in English, lacking full text, or not directly relevant to the topic were excluded. Titles and abstracts were screened initially, followed by full-text evaluation. Reference lists of included studies were also manually searched to identify additional relevant publications. The study selection process is presented using a PRISMA flow diagram.

Brief scenario

*E. coli* can cause serious extraintestinal and gastrointestinal (diarrheal) diseases [3]. Hemolytic-uremic syndrome (HUS), septicemia, pneumonia, meningitis, kidney and bladder infections, diarrhea, and dysentery are among the complications that *E. coli* strains can cause. However, diverse strains with distinct virulence genes cause different symptoms. Most *E. coli* strains in the colon are non-virulent, although some possess virulence factors that cause disease [4]. All members of the *Enterobacteriaceae* are rod-shaped, Gram-negative, facultative anaerobes that primarily colonize both human and animal gastrointestinal tracts. They are usually the cause of community-associated and healthcare-associated infections. Gram-negative bacterial infections were previously successfully treated with the majority of first-line, inexpensive antimicrobial medications, including penicillin and first and second-generation cephalosporins. However, drug-resistant organisms develop resistance to first-line antibiotics, thereby necessitating the use of second-line agents such as third- and fourth-generation cephalosporins. Carbapenems, including ertapenem, imipenem, meropenem, and doripenem, have demonstrated effectiveness in treating severe diseases caused by these multidrug-resistant (MDR) *Enterobacteriaceae*. However, Carbapenem-resistant *Enterobacteriaceae* (CRE) have become a significant health risk in the community [5].

Carbapenem resistance is significantly associated with high rates of morbidity, mortality, and treatment failure in numerous infectious illnesses. There are a few antibiotics that are active against bacteria that produce carbapenemase. At present, CRE is an issue that affects entire communities and nations rather than only particular individuals [5]. Many of the resistance genes found in *E. coli* isolates during the past few decades were acquired by horizontal gene transfer [2].

Epidemiology and spread

There are various types of substrates on which *E. coli* can survive. The habitat of *E. coli* is determined by the availability of nutrients in the host species' intestines [6]. The primary habitat for *E. coli* is the gastrointestinal tract of human beings and other warm-blooded animals. The physical characteristics of these habitats, such as temperature and nutritional availability, differ considerably. In the primary habitat, the temperature is generally constant at about 37°C, while in the secondary habitat, annual average temperatures can fluctuate significantly, ranging from below freezing (0°C) to about 18°C or more. Furthermore, the secondary environment (such as deep soil, sediment, and water resources) can be either aerobic or anaerobic, whereas the primary habitat is anaerobic. Meanwhile, there are typically fewer nutrients found in the secondary environment, particularly in soil and sediment, which are constantly deficient in nutrients when it comes to bacterial growth. Nutrient levels in water can range from high (receiving waters in agricultural areas, for example) to low (open ocean). This is in contrast to the nutritional circumstances of the primary habitat (i.e., the colon), which are consistently high and promote rapid bacterial growth. As a result, the growth and survival of *E. coli* will usually be more difficult in the secondary habitat [7].

The gastrointestinal tracts of humans and animals serve as the primary habitat for the ubiquitous Gram-negative bacterium, *E. coli*. Although most of the strains are commensals, a few have developed pathogenic characteristics that cause a wide range of intestinal and extraintestinal diseases. It is a main cause of bloodstream infections, respiratory infections, and urinary tract infections (UTIs) [8]. The clinical burden of *Escherichia coli* infections is particularly highest in developing countries, especially in regions such as sub-Saharan Africa, where inadequate infection prevention measures, inappropriate antibiotic use, and limited access to diagnostic facilities contribute to the emergence and spread of resistance [9]. Meanwhile, the bacteria play an important role in the development of AMR in gram-negative infections, which include resistance to aminoglycosides, fluoroquinolones, and broad-spectrum beta-lactams. Both community-acquired and nosocomial infections are connected to rising rates of ESBL-producing *E. coli* and, more recently, carbapenem-resistant strains, which provide serious difficulties for therapeutic care [8]. The most common bacteria found in blood, urine, cerebrospinal fluid, and respiratory specimens were *E. coli*. Approximately 99,000 culture-positive isolates from all clinical samples from tertiary care institutions nationwide were recorded by the Indian Council of Medical Research (ICMR), Antimicrobial Resistance Surveillance Network (AMRSN) in 2023 [8].

Both established and emerging drug-resistant bacteria are spreading around the world because of globalization and the extraordinary increase in human movement due to travel, migration, healthcare transfers, and conflict. National boundaries do not limit these infections. Visitors to areas with an elevated risk of antibiotic-resistant diseases can colonize and unintentionally spread these bacteria back to their home country. Tourists from high-prevalence areas can also introduce resistant strains into host populations. Transmission of multi-resistant superbugs from the patient's country of origin is a concern for medical tourism. [10]. Treatment failures, extended hospitalizations, high medical expenses, and socioeconomic burdens on healthcare facilities are all outcomes of these infections. Several factors contribute to the rise of antimicrobial resistance (AMR) in India. These include over the counter (OTC) dispensing of antibiotics without prescription, the reuse of antibiotics based on previous prescriptions, and gaps in continuing medical education among healthcare professionals. Additionally, inadequate antimicrobial stewardship programs and limited resources in secondary healthcare settings further exacerbate the problem. Misuse of antibiotics and a lack of monitoring are also significant contributors to the development and spread of AMR. The ICMR established evidence-based therapy guidelines to address this expanding problem. Antimicrobial susceptibility testing (AST) must be done thoroughly before beginning antimicrobial medication, particularly to determine the minimum inhibitory concentration [11].

Mechanisms of resistance

It was once believed that humans had effectively controlled microbial infections following the discovery of antibiotics; however, resistance rapidly emerged [12]. The mechanism of antibiotic activity can be used to categorize antimicrobial agents. The primary categories include substances that stop the production of cell walls, depolarize the cell membrane, prevent the synthesis of proteins, stop the synthesis of nucleic acids, and prevent bacterial metabolic pathways. Additionally, bacteria may acquire the resistance genes from similar species; the species and acquired genes will determine the level of resistance [12].

Natural or Intrinsic Resistance

Natural or intrinsic resistance is a characteristic typical of all members of a species, unaffected by prior antibiotic exposure. Decreased permeability of the outer membrane, particularly the lipopolysaccharide in gram-negative organisms, and the inherent activity of efflux pumps are the most prevalent bacterial processes implicated in intrinsic resistance [12].

Acquired Resistance

Bacteria acquire genetic material through three primary mechanisms: transformation, transposition, and conjugation, which enable bacteria to acquire genetic material that confers resistance. Additionally, the bacterium undergoes chromosomal DNA mutations. The average rate of mutation in bacteria is one every 106-109 cell divisions, and the majority of such mutations are damaging to the cell. Even very low or sub-inhibitory antimicrobial concentrations can select for hypermutable strains of bacteria (increasing the rate of mutation), enhance the potential to become resistant to other antimicrobial drugs, and facilitate the transfer of mobile genetic elements (mobilome). These effects can result in the selection of extreme resistance in subsequent generations of bacteria [12].

Mechanisms of AMR

Mechanisms of AMR can be categorized into four major types: (i) reduced drug intake, (ii) alteration in drug target, (iii) neutralizing drug, and (iv) active efflux pumps. Intrinsic resistance may make use of reduced intake, drug neutralization, and efflux pumps; acquired resistance mechanisms used may be alteration in the drug target, drug neutralization, and efflux pumps [12].

Reduced Drug Intake

Bacteria naturally differ in their capacity to reduce the intake of antimicrobial drugs. In Gram-negative bacteria, the functions and structure of the lipopolysaccharide layer act as a barrier against specific kinds of molecules. Because of this, such bacteria are naturally resistant to specific classes of effective antimicrobial agents. Substances frequently reach the bacterium through porin channels in bacteria with broad outer membranes. Hydrophilic compounds can typically enter gram-negative bacteria through their porin channels. A reduction in the quantity of porins and mutations that alter the porin channel's selectivity are the two primary ways that porin alterations can restrict medication absorption. Members of *Enterobacteriaceae* can develop resistance because of a decrease in porins, which reduces antibiotic entry into the bacterial cell. These bacteria reduce the quantity of porins as a defense against carbapenems. The development of a biofilm protects harmful organisms from both antimicrobial agents and the human immune system. Antimicrobial drugs find it challenging to penetrate the bacteria due to the thick, sticky quality of the biofilm matrix, which comprises proteins, DNA, and polysaccharides from the resident bacteria. Therefore, substantially larger medication concentrations are required for them to be effective [12].

Alteration in Drug Target

Antimicrobial medications may target a variety of bacterial cell components, and the bacteria may alter these targets to develop resistance against the drugs. Changes in the structure of penicillin-binding proteins (PBPs) are one of the reasons for resistance to the widely used β-lactam antibiotics. Peptidoglycan is formed in the cell wall by PBPs. The number of drugs capable of binding to the specific target is affected by variations in the quantity of PBPs. Drug binding may be reduced or completely inhibited by a structural alteration. Due to these structural alterations, the bacteria develop resistance to such drugs. Resistance to drugs that disrupt the synthesis of nucleic acids (fluoroquinolones) is caused by alterations in DNA gyrase (gram-negative bacteria, e.g., gyrA). The drug's capacity to attach to gyrase and topoisomerase is reduced due to such alterations, which modify their structures [12].

Neutralizing Drug

Bacteria can inactivate drugs in two primary ways: either by transferring a chemical component of the drug or by actually degrading the drug. A large class of enzymes that hydrolyze drugs is called "β-lactamases." Tetracycline is another medication that may be rendered inactive by hydrolysis through the *tetX* gene. Acetyl groups, phosphoryl groups, and adenyl groups are the most frequently transferred chemical groups used in drug inactivation. Numerous transferases have been found. Acetylation is the most adaptable process that is proven to be successful against fluoroquinolones, streptogramins, aminoglycosides, and chloramphenicol. It is recognized that the aminoglycosides are the main targets of phosphorylation and adenylation [12].

Efflux Pump

Efflux pump genes are chromosomally encoded in bacteria. High-level resistance is typically caused by a mutation that alters the transport channel in response to certain environmental stimuli or the existence of an appropriate substrate. The majority of bacteria have an extensive range of efflux pumps. Based on their energy and structure source, bacteria have 5 main families of efflux pumps [12]. Those families of efflux pumps are described in Table 1.

**Table 1:** Families of efflux pumps ABC: ATP-Binding Cassette; MATE: Multidrug and Toxic Compound Extrusion; SMR: Small Multidrug Resistance; MFS: Major Facilitator Superfamily; RND: Resistance-Nodulation-Cell Division; ATP: Adenosine Triphosphate; *E. coli*: *Escherichia coli*

Family	Energy Source	Functions	Example
ATP-Binding Cassette (ABC)	ATP hydrolysis (Chemical energy)	Transport amino acids, drugs, ions, polysaccharides, proteins, and sugars	Found in *Vibrio* *cholerae*; capable of transporting fluoroquinolones and tetracycline
Multidrug and Toxic Compound Extrusion (MATE)	Sodium ions	Efflux cationic dyes, fluoroquinolone drugs, and some aminoglycosides	—
The Small Multidrug Resistance (SMR)	Proton-motive force	Efflux mainly lipophilic cations	Found in chromosomal DNA, on plasmids and transposable elements; seen in *E. coli*
Major Facilitator Superfamily (MFS)	Proton-motive force and sodium ions	Transport of anions, metabolites, and sugars	*E. coli* having separate MFS pumps for macrolides; nearly 50% of the efflux pumps in *E. coli* are MFS pumps
Resistance-Nodulation-Cell Division (RND)	Proton exchange	Multi-drug transporters; export antibiotics, metals, and solvents	AcrAB-TolC pump in *E. coli*, which confers resistance to penicillins

β-lactamases

**Figure 2:**
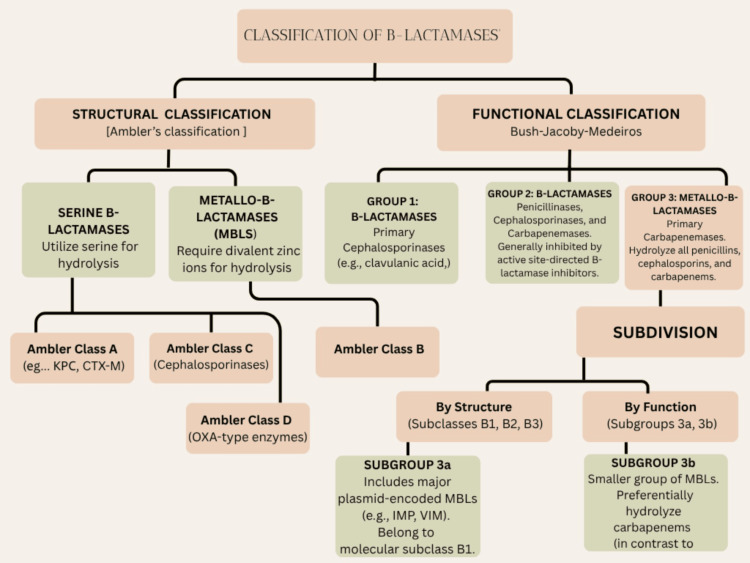
Classification of β-lactamases Source: Authors’ own work based on reviewed literature.

The β-lactam drugs are a frequently utilized class of antibacterial agents. The classification of β-lactamase is described in Figure 2. A four-sided β-lactam ring serves as the common core structure for the drugs in this group. Three general mechanisms lead to resistance to β-lactam drugs: (i) blocking the drug and target PBPs from interacting, often by altering the drug's capacity to bind to the PBPs; (ii) the existence of efflux pumps capable of extruding β-lactam drugs; (iii) hydrolysis of the drug by β-lactamase enzymes. β-lactamases render these drugs inactive by hydrolyzing the amide bond of the β-lactam ring, preventing the drug from binding to target PBPs [12].

Certain β-lactamases, particularly carbapenemases, have evolved the ability to hydrolyze carbapenems. The enzymes primarily responsible for resistance include New Delhi metallo-β-lactamase (NDM) and *Klebsiella pneumoniae carbapenemase* (KPCs), which are types of carbapenemases. The *AmpC* gene chromosomally encodes the first β-lactamase to be identified, which came from *E. coli*. The most prevalent CREs are VIM-1 (Verona integron-encoded MBL) and IMP-1 (imipenem resistance). Recently, a novel MBL was discovered, mostly in *E. coli* strains. It is known as New Delhi metallo-β-lactamase-1 (NDM-1). A total of 71% of hospital deaths have been linked to infections brought on by CRE strains [12].

Clinically important β-lactamases include ESBL, *AmpC*, and carbapenemases.

Extended-Spectrum β-Lactamases (ESBLs)

Such enzymes have the ability to hydrolyze all penicillins, cephalosporins (1st, 2nd, 3rd, and 4th generations), and aztreonam, with the exception of carbapenems and cephamycins, which are produced by gram-positive and gram-negative bacteria. However, resistance to carbapenems may arise from both loss of porin channels and hyperproduction of ESBLs. Clinically significant ESBLs include TEM (Temoniera) β-lactamase, SHV (sulfhydryl reagent variable) β-lactamase, CTX-M (Cefotaxime-Munich) β-lactamase, and OXA (oxillinase) β-lactamase [13].

AmpC β-Lactamases

These enzymes are resistant to β-lactam/β-lactamase inhibitors, cephamycins, penicillins, and 1st, 2nd, and 3rd generation cephems, yet not to carbapenems and 4th generation cephems. However, resistance to carbapenem may arise from hyperproduction of *AmpC* β-lactamases with porin loss. The three modes of *AmpC* resistance are plasmid-mediated *AmpC* genes, stable chromosomal de-repression, and inducible chromosomal resistance [13].

Carbapenemase

Almost all β-lactam antibiotics, such as carbapenems, broad-spectrum penicillins, cephalosporins, and cephamycins, are hydrolyzed by these enzymes. The following are various types of carbapenemases [13].

Group 2F or Class A Serine Carbapenemases

Plasmid-encoded KPC-type β-lactamases are the most common type of this group. Traditional β-lactamase inhibitors like clavulanate inhibit it weakly, whereas newer inhibitors like avibactam and vaborbactam are more effective against Class A carbapenemases. They have low-level carbapenemase activity, but they are able to develop high-level carbapenem resistance when paired with other resistance mechanisms, such as porin loss. The other, less prevalent classes, Guiana extended-spectrum (GES), non-metallo carbapenemase (NMC), imipenem-hydrolyzing (IMI), and Serratia marcescens enzyme (SME), are examples of class A carbapenemases [13].

Group 2DF or Class D Serine Carbapenemases

They are also known as OXA-type carbapenemases. Normally, the *Acinetobacter baumannii* complex contains mostly OXA-type enzymes. OXA-51, a chromosomal OXA-type carbapenemase present in every strain of *A. baumannii*. *A. baumannii* contains three additional OXA-type carbapenemase gene clusters: OXA-23-like, OXA-24-like, and OXA-58. OXA-48 is a common OXA-type carbapenemase in *Enterobacterales*. OXA-48 and its related derivatives, especially OXA-181 and OXA-232, are more proactive against imipenem than meropenem and have low-level carbapenemase activity. However, it can lead to high-level carbapenem resistance when paired with other resistance mechanisms, such as porin loss [13].

Group 3 or Class B Metallo-ẞ-Lactamases (MBL)

The three types of acquired Class B MBL-NDM, IMP, and VIM, as well as the SIM, GIM, and SPM types, are frequently included. With the exception of aztreonam, these enzymes have strong hydrolytic activity against all ẞ-lactams. The ion chelator ethylene diamine tetra-acetic acid (EDTA) inhibits them, although ẞ-lactamases do not. The MBL genes are found on specialized genetic elements that aid in the acquisition and spread of the resistance determinant, such as integrons, transposons, and plasmids [13].

Clinical impact

The tremendous problem of resistance to antibiotics that we currently face is a result of improper stewardship of antimicrobial agents. Overuse of antimicrobial medications by both humans and animals, in addition to improper prescribing of antimicrobial therapy, are factors that have contributed to the growing problem of resistance. Physicians may overuse several popular antimicrobial medicines because they choose medications based on their low cost and low toxicity. Additionally, antimicrobial medications may be prescribed incorrectly, such as when a broad-spectrum medication is first prescribed that is either unnecessary or subsequently shown to be ineffective against the organisms causing the infection [12].

AMR is associated with millions of deaths annually worldwide; however, precise estimates vary regarding the attributable mortality of drug-resistant versus drug-sensitive infections. The most complete worldwide assessment of the impact of AMR, spanning 204 countries, 23 diseases, and 88 pathogen-drug combinations, was recently released by the Antimicrobial Resistance Collaboration. After analyzing data from surveillance programs, healthcare systems, and systematic literature reviews, the partnership estimated that in 2019, approximately 4.95 million deaths were associated with bacterial antimicrobial resistance (AMR), of which 1.27 million were directly attributable to it. With 829,000 AMR-related fatalities and 219,000 AMR-attributable deaths, *E. coli* was the leading culprit pathogen [14].

According to a review of AMR 2016, 10 million deaths will be associated with AMR by 2050 [9]. In both developed and developing nations, AMR raises health care expenses, hospital stays, morbidity, and mortality. In order to demonstrate the clinical impact of resistant bacteria in WHO regions worldwide, the WHO released the first global surveillance report on resistance of antibiotics in 2014. According to this assessment, *E. coli* in five of the six WHO regions exhibited more than 50% resistance to fluoroquinolones and third-generation cephalosporins [15]. Since 2017, over 80% of approved antibiotics for CRE infections have shown some degree of novel efficacy; these drugs are variants of well-known antibiotic families that contain a number of resistance mechanisms. As a result, this increases the difficulties and complexity of antimicrobial resistance. Recent years have seen the introduction of new antibiotics and innovative combination drugs, such as β-lactam inhibitors with carbapenems or cephalosporins, which primarily target Class A and certain Class D enzymes [16].

The review on AMR issued a research paper on quick diagnostics and suggests promoting "new, rapid diagnostics to cut unnecessary use of antibiotics." Automated phenotypic testing, biomarker tests, and point-of-care tests are examples of quick diagnostics; however, definitions vary based on application and time to result (TTR, time from sample collection to results returned). Several international studies have revealed that diagnostics, especially quick diagnostics, shorten the time it takes to start the best antibiotic treatment. Additionally, rapid diagnoses improve patient clinical results, lower mortality, and improve financial outcomes, such as shorter hospital stays and lower medical expenses. Rapid diagnostic testing has a significant influence on several aspects of antimicrobial stewardship programs in addition to lowering antimicrobial resistance [17]. Antibiotic resistance has a direct effect on people's health. It limits our ability to develop an effective treatment plan to end infectious illnesses and reduce morbidity and mortality rates [11].

Public health and research

AMR, which is anticipated to lead to more deaths than cancer by 2050, is generally acknowledged as a danger to world health. The World Health Organization's Global Action Plan on AMR serves as a major model for the National Action Plan that the Indian government has created to combat AMR. In 2015, the World Health Assembly approved WHO’s Global Action Plan on AMR, which served as a major model for the NAP-AMR. This review examines the implementation, scope, and progress of the National Action Plan on Antimicrobial Resistance (NAP-AMR) in India, with a focus on the human health sector. Although the NAP-AMR effectively reflects the Global Action Plan and outlines challenging targets, we find that progress has been impeded by weak multisectoral coordination, poor enforcement, and a lack of funding allocation across states. Monitoring the extent and scope of AMR is made even more difficult by the lack of standardized surveillance data, which primarily comes from published studies of healthcare-associated infections in inpatient settings, scoping reports, prospective studies, and point prevalence surveys at specific large hospitals [18]. The current National Action Plan on Antimicrobial Resistance (NAP-AMR) in India demonstrates alignment with the Global Action Plan on Antimicrobial Resistance developed by the World Health Organization, particularly in areas such as surveillance strengthening, antimicrobial stewardship, infection prevention and control, and public awareness initiatives. GAP's main goals are included in the plan, along with an extra goal of bolstering India's leadership in AMR [19].

The National Action Plan on Antimicrobial Resistance (NAP-AMR) outlines a comprehensive strategy centered on six strategic pillars: (i) improving awareness through education and training; (ii) strengthening evidence through systematic surveillance of resistance patterns; (iii) reducing infection incidence via rigorous prevention and control measures; (iv) ensuring the rational use of antimicrobials through stewardship across all sectors; (v) accelerating investment in research, diagnostics, and novel treatments; and (vi) exerting national and global leadership through multi-sectoral collaboration [19]. AMR has risen sharply in recent years due to a marked slowdown in the development of new antimicrobial agents. Although they have limited choices for treatment for CRE infections, they are challenging to manage and cause a significant economic and health burden. This emphasizes the need for new and creative treatment alternatives in addition to effective preventative and control strategies [16].

Alternative methods to antibiotics

Combination Therapy

Combination therapy involves the use of two or more antibiotics according to their susceptibility patterns in order to maximize their synergistic effects. The inhibition of a mutual target and distinct routes, the inhibition of distinct targets and the same pathway, and the inhibition of the same target and the same pathway are three possible mechanisms of this strategy. Bactrim, a combination of trimethoprim and sulfamethoxazole, is an example of a combination medication that inhibits two distinct targets in the same pathway. Each of Bactrim's ingredients blocks a distinct stage of the same pathway's folic acid production. They specifically target dihydropteroate synthase and dihydrofolate reductase [20].

Drug-Adjuvant Combinations

Adjuvants are compounds that either directly target resistance mechanisms, indirectly increase the antibacterial effects by blocking efflux pumps, or interfere with bacterial signaling pathways to increase the antibacterial activity of an antibiotic. Augmentin, a medication-adjuvant combination made up of clavulanic acid and amoxicillin, is frequently administered [20].

Antimicrobial Peptides (AMPs)

Short, positively charged peptides called AMPs are part of the innate immune system of higher animals, plants, insects, and prokaryotes. AMPs target the bilayer structures found in the cell walls of gram-negative and gram-positive bacteria to produce their antibacterial actions. AMPs carry a positive charge, which makes them interact with both the negatively charged lipoteichoic acid in the membranes of Gram-positive bacteria and the negatively charged polysaccharides in the cell membranes of Gram-negative bacteria. Because bacteria rarely become resistant to this method, AMPs are beneficial antimicrobials. In addition, because antimicrobial peptides have special properties, resistance is uncommon. Diverse mechanisms of action are made possible by each AMP's unique structure, peptide-to-lipid ratio, and lipid membrane characteristics [20].

Monoclonal Antibodies (MABs)

B cells create MABs, which act by specifically targeting antigens. However, the FDA has authorized only three monoclonal antibodies. These consist of obiltoxaximab, raxibacumab, and bezlotoxumab. By neutralizing secretory toxins, these MABs demonstrate their antimicrobial properties [20].

Bacteriophages

Bacteriophages are viral entities that invade bacteria and may be genetically altered for a variety of antimicrobial uses. Other antibacterial agents do not have the same molecular effects as bacteriophages. Adsorption to host surface receptors via a lock-and-key interaction is the initial stage of bacteriophage infection. The phage transfers its genetic material into the bacterial host cell upon adsorption. Peptidoglycan in the bacterial cell wall is broken down by lytic phages, which replicate inside the host bacterial cells and create lytic enzymes called virolysins. Ply6A3, an endolysin, for instance, has shown significant antibacterial activity against *E. coli* [20].

Probiotics

The synthesis of antimicrobial bacteriocins, improvement of mucosal barriers to prevent bacterial attachment and entry into the mammalian gut, removal of toxins, restoration of gut dysbiosis, and induction of immunomodulation by promoting protective cytokines and suppressing pro-inflammatory cytokines are the mechanisms by which these microorganisms produce antibacterial effects [20].

Nanomaterials and Nanoparticles

Numerous infections, particularly those brought on by MDR strains, can be treated using nanoparticles (NPs) and nano-drug carriers. Nanoparticle processes may be divided into two categories: enhancing the effects of current antibiotics and producing novel bactericidal effects that are independent of these antibiotics. Many drug-resistant bacterial species, such as *S. aureus*, *E. faecium*, *E. faecalis*, *E. coli*, and *V. cholerae*, were successfully combatted by chemically produced gold nanoparticles. Zinc oxide and titanium dioxide have shown antibacterial activity against *E. coli* [20].

Cannabinoids

Cannabinoids fall into one of two categories: endogenous or exogenous. Cannabinoid 1 and cannabinoid 2 receptors are affected by both endogenous and exogenous cannabis. Target pathogens like *E. coli* were sensitive to the antibacterial actions of cannabinoids [20].

## Conclusions

The emergence of carbapenemase-producing *Escherichia coli* (*E. coli*) poses a significant threat to global public health. Antibiotic misuse and overuse, improper infection control practices, and a lack of diagnostic resources have all contributed to the rapid development of resistant strains, particularly in developing nations. Carbapenem-resistant *E. coli* severely limited treatment choices, resulting in higher rates of treatment failure, elevated mortality, longer hospital stays, and increased medical expenses. This challenge is further intensified by the ability of these bacteria to acquire resistance genes through horizontal gene transfer, enabling rapid spread across different populations and environments. Improved infection control practices, strengthened antimicrobial stewardship programs, and rational antibiotic use are essential to combat antimicrobial resistance. Key strategies include strict adherence to hand hygiene protocols, implementation of hospital infection prevention and control (IPC) measures, routine surveillance of antimicrobial resistance patterns, and enforcement of antibiotic prescribing guidelines. Additionally, antimicrobial stewardship programs should incorporate audit and feedback mechanisms, formulary restrictions, and clinician education to optimize them. Furthermore, appropriate therapy can be guided by an early and accurate diagnosis made with fast diagnostic techniques. Research on novel therapeutic approaches, such as combination medications, bacteriophages, and antimicrobial peptides, is also essential.
